# Ion Dynamics in Nanocrystalline Li_2_S‐LiI – on the Influence of Local Disorder on Short‐Range Hopping and Long‐Range Ion Transport

**DOI:** 10.1002/smsc.202400199

**Published:** 2024-07-30

**Authors:** Anna Jodlbauer, Katharina Hogrefe, Bernhard Gadermaier, H. Martin R. Wilkening

**Affiliations:** ^1^ Institute of Chemistry and Technology of Materials Graz University of Technology Stremayrgasse 9 A‐8010 Graz Austria

**Keywords:** composites, conductivity, ion dynamics, lithium sulfide, nuclear magnetic resonance

## Abstract

The enormous interest in developing powerful Li‐based batteries leads to a boost in materials research. Though Li–sulfur batteries offer very high energy densities, the nature of Li‐ion dynamics in the final discharge product ${\text{Li}}_{2}S$ has not been fully understood yet. While nanocrystalline ${\text{Li}}_{2}S$ shows enhanced ion dynamics compared to its coarse‐grained counterpart, the interaction of ${\text{Li}}_{2}S$ with another binary such as LiI seems to be rather unexplored. Herein, an equimolar mixture of ${\text{Li}}_{2}S$ and LiI is treated in a high‐energy ball mill, and both the overall and local structural changes are studied by X‐ray powder diffraction and 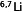 nuclear magnetic resonance (NMR), respectively . Besides the formation of amorphous regions, evidences are found for the generation of anion‐mixed sites that give rise to facile Li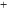 exchange on the 2D exchange NMR timescale. Compared to a coarse‐grained reference sample, the overall (bulk) ionic conductivity of nanocrystalline ${\text{Li}}_{2}S$‐LiI increases by two orders of magnitude. Besides the anion‐mixing effect, this increase benefits from nanosize effects that include the formation of defect‐rich interfacial regions. NMR relaxation measurements fully support this result and reveal heterogeneous dynamics with lower activation energies for both the localized hopping processes and long‐range ion transport in nm‐sized ${\text{Li}}_{2}S$‐LiI.

## Graphical abstract

Mixtures of Li_2_S and LiI, if present in a nanocrystalline form, show ionic conductivities exceeding that of nanostructured (single phase) Li_2_S by two orders of magnitude. Nuclear magnetic resonance (NMR) spin‐lattice relaxation measurements sense the same huge change in Li diffusivity. Herein, 1D and 2D Li exchange NMR is used to understand how Li^+^ exchange occurs between the magnetically distinct Li environments.

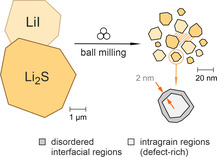

## 1 Introduction

The annual increase in worldwide energy usage is accompanied by a corresponding raise in greenhouse gas emissions. Currently, our energy infrastructure still depends on nonrenewable and finite fossil fuels rather than on sustainable resources. To mitigate greenhouse gas emissions and to enable the transition to a more sustainable energy system, the utilization of effective electrochemical energy storage solutions such as Li‐ion batteries will be pivotal.^[^ ^1^, ^2^, ^3^ ^]^

Hence, there is a pressing requirement for materials development that allow realizing battery systems capable of storing high amounts of energy, which can be used in both the transport sector and stationary energy storage for the grid.^[^ ^4^, ^5^, ^6^, ^7^ ^]^ Especially high energy densities in Li‐based batteries could be reached when taking advantage of the reaction of Li with elemental sulfur; in such systems, energy densities as high as 1167 mAhg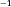 are achievable.^[^ ^8^ ^]^ The basic principle of Li–sulfur batteries is based on the formation of ${\text{Li}}_{2}S$ during discharge. For the reverse reaction, that is, charging the Li–S battery, ${\text{Li}}_{2}S$ needs to be electrochemically decomposed into Li and S again. The overall performance of the cell, that is, discharge capacities and the cycle life, does sensitively depend on the electrochemical properties of the active material.^[^ ^9^ ^]^ Specifically, it also depends on the ability of ${\text{Li}}_{2}S$ to conduct Li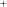 ions (and electrons). Unfortunately, ${\text{Li}}_{2}S$ shows a very poor overall conductivity of only $10^{-10}\;S\;{\text{cm}}^{-1}$ at 80 °C.^[^ ^10^ ^]^ The discharge capacities and the cycle life of such type of batteries are limited by the low electrochemical conductivity of the active material ${\text{Li}}_{2}S$.

Unexpectedly, only few fundamental studies can be found that focus on electrochemical properties^[^ ^9^ ^]^ and the characterization of transport properties in ${\text{Li}}_{2}S$ in its different morphologies, particularly when present in a nanocrystalline form.^[^ ^10^, ^11^, ^12^ ^]^ Several studies and attempts can be found in literature that try to modify and increase the ionic conductivity of ${\text{Li}}_{2}S$. In particular, ${\text{Li}}_{2}S$ and LiI have been mixed in different molar ratios to manipulate overall Li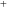 transport.^[^ ^8^, ^9^, ^13^ ^]^ In general, highly conducting electrolytes being composed of abundant elements are also needed for the development of Li‐based solid‐state batteries with classical cathode and anode materials.^[^ ^14^, ^15^, ^16^ ^]^ As an example, Hakari et al.^[^ ^8^ ^]^ tried to enhance the transport properties of ${\text{Li}}_{2}S$ by improving its ionic conductivity through crystal engineering. They replaced the S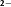 anions by the larger monovalent iodine ions resulting in distortions in the crystal lattice. The authors prepared (100−x)${\text{Li}}_{2}S$:*x*LiI (mol % 0≤x≤25) and showed that a LiI content of up to x=20 led to a significant improvement in performance^[^ ^17^ ^]^ and ionic conductivity at room temperature ($2.2\times 10^{-6}\;S\;{\text{cm}}^{-1}$) which exceeds that of pulverized ${\text{Li}}_{2}S$. Similarly Hakari et al.^[^ ^13^ ^]^ tried to improve the utilization of ${\text{Li}}_{2}S$ by investigating the influence of ionic conductivity and the type of ${\text{Li}}_{2}S$ solid solutions on the overall cell performance. For this purpose, ${\text{Li}}_{2}S$‐LiX (X = Cl, Br, and I) solid solutions were prepared; 80${\text{Li}}_{2}S$‐20LiI yielded the most promising results with a ${\text{Li}}_{2}S$ utilization of 95 % due to the increased number of electrochemical reaction sites.^[^ ^13^ ^]^ Further work showed that the initial ${\text{Li}}_{2}S$‐LiI solid solution decompose yielding a material with LiI domains; these domains were suggested to be responsible for the high reversible charge capacity observed.^[^ ^9^ ^]^

In literature, we also find attempts to directly prepare ${\text{Li}}_{3}\text{SI}$ from equimolar mixtures of ${\text{Li}}_{2}S$ and LiI. The Ag‐bearing counterpart ${\text{Ag}}_{3}\text{SI}$ crystallizing with antipervoskite structure is known as a very fast ionic conductor reaching room temperature values as high as 38 mS cm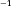 if the α*‐phase is considered.^[^ ^18^, ^19^, ^20^ ^]^ Historically, extremely high Ag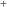 conductivity was observed already in the 1910s for a series of Ag‐bearing materials, including the most prominent example *α*‐AgI^[^ ^21^, ^22^ ^]^ and others such as ${\text{Ag}}_{2}X$ (X = S, Se, Te) and ${\text{MAg}}_{4}I_{5}$ (M = Rb, K, ${\text{NH}}_{4}$).^[^ ^23^, ^24^, ^25^, ^26^ ^]^ Coming back to ${\text{Ag}}_{3}\text{SI}$, there is no clear evidence yet that the Li counterpart ${\text{Li}}_{3}\text{SI}$ does indeed exist. Yin et al.^[^ ^27^ ^]^ studied the synthesis mechanism of ${\text{Ag}}_{3}\text{SI}$ by in situ X‐ray measurements to use these information for a targeted preparation of ${\text{Li}}_{3}\text{SI}$. Unfortunately, the preparation remained unsuccessful because LiI and ${\text{Li}}_{2}S$, in contrast to the situation for ${\text{Ag}}_{3}\text{SI}$, do not from a common bcc close‐packing of spheres.^[^ ^27^ ^]^ In contrast, Rajagopa et al.^[^ ^28^ ^]^ report on the formation of solid‐solution‐like ${\text{Li}}_{3}\text{SI}$ as suggested by X‐ray diffraction and Raman spectroscopy. The authors report on conductivities of $10^{-6}\;S\;{\text{cm}}^{-1}$.^[^ ^28^ ^]^ In a succeeding work, they mixed ${\text{Li}}_{2}S$ and LiI in molar ratios of 1:1, 2:1, 3:1, and 4:1. A maximum in conductivity ($3.1\times 10^{-5}\;S\;{\text{cm}}^{-1}$) is reported for the 2:1 mixture.^[^ ^28^ ^]^

Here, following the idea to prepare ${\text{Li}}_{3}\text{SI}$ from equimolar mixtures of ${\text{Li}}_{2}S$ and LiI, we used high‐energy ball milling to mix the two binaries. Afterward, we exposed these nanocrystalline mixtures to different annealing procedures to study the change in both local structure and ion dynamics on different length scales by means of a series of techniques such as X‐ray diffraction, broadband conductivity spectroscopy, electric modulus measurements as well as time‐domain and (1D, 2D) high‐resolution 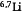 nuclear magnetic resonance (NMR) spectroscopy. The sample directly obtained by high‐energy ball milling shows a room temperature ionic conductivity exceeding that of the fully annealed sample by two orders of magnitude. It is also higher than that found for ${\text{Li}}_{2}S$ thin films.^[^ ^11^, ^12^ ^]^ Such higher conductivity might be beneficial for Li–sulfur batteries if one would manages to produce I‐containing ${\text{Li}}_{2}S$ as a nanostructured discharge compound. Jumping of the Li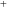 ions between ${\text{Li}}_{2}S$ and LiI environments, as directly visualized by 2D 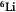 exchange NMR, seems to be responsible for the increase observed for the nanocrystalline sample. Besides this mixing effect, defects in the interfacial regions and (nontrivial) nanosize effects including the role of space charge regions might contribute to enhanced ion dynamics in these nanoionics.^[^ ^29^, ^30^, ^31^ ^]^ The latter effects are often found in nanocomposites with conductor–insulator interfaces^[^ ^30^, ^31^, ^32^, ^33^, ^34^, ^35^, ^36^, ^37^ ^]^ or even glass/crystal interfaces.^[^ ^38^ ^]^

## 2 Results

### 2.1 Structural Properties: XRD and MAS NMR

**Figure 1:**
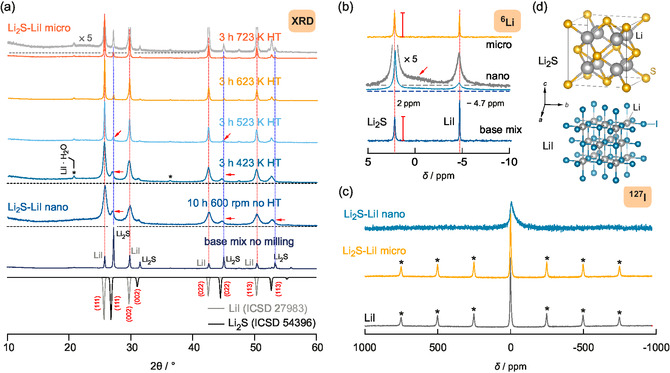
a) X‐ray powder diffractograms of the samples studied. To obtain the so‐called base mix, ${\text{Li}}_{2}S$ and LiI were mixed by hand in a mortar for 2 min. The nanocrystalline sample was prepared by high‐energy ball‐milling using a planetary mill. Subsequently, this sample was annealed (or heat‐treated, HT) at different temperatures ranging from 423 to 723 K; the sample annealed at 723 K represents the sample referred to as microcrystalline ${\text{Li}}_{2}S$‐LiI. At the bottom, the reference XRD patterns of LiI (gray, ICSD no. 27 983) and ${\text{Li}}_{2}S$ (black, ICSD no. 54 396) are included. Asterisks mark reflections of $\text{LiI}\cdot H_{2}O$ showing up as a minor impurity. b) 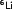 MAS NMR spectra acquired at 73.6 MHz and at a spinning speed of 25.0(1) kHz. c) 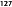
I (100.1 MHz) MAS NMR spectra recorded at room temperature and again a spinning speed of 25.0(1) kHz. For comparison, the 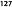
I MAS NMR spectrum of the starting material LiI is shown as well. It serves as a reference for the chemical shift range ($\delta_{\text{iso}}{(}^{127}I\;\text{in}\;\text{LiI})=0\;\text{ppm}$). d) Illustration of the crystal structures ($Fm\bar{3}m$) of cubic ${\text{Li}}_{2}S$ and cubic LiI.

X‐ray powder diffraction and magic angle spinning (MAS) NMR, see **Figure** **1**a and Figure 1b, have been used to characterize structural features of the composites prepared. In Figure 1a, the X‐ray powder diffraction patterns of the base mix, the nanocrystalline, and the annealed samples are shown. The base mix was prepared by simply mixing the binary starting materials by hand in a mortar. Milling in a planetary mill (10 h, 600 rpm) yields nanocrystalline ${\text{Li}}_{2}S$‐LiI which is characterized by broad reflections due to nm‐sized crystallites and strain/stress introduced. Annealing this sample at the temperatures and durations indicated resulted in a series of samples with again sharper reflections. The sample annealed at 723 K is called the microcrystalline sample.

For comparison, in Figure 1a, we included the reference patterns of the starting materials LiI and ${\text{Li}}_{2}S$ as found in the International Crystal Structure Database (ICSD). As it is clearly seen, milling the mixture of ${\text{Li}}_{2}S$ and LiI causes the broadening of all reflections and a decrease in the intensity of those reflections belonging to the softer sulfide, see the signals at 25.6° and 27°, respectively. Hence, the decrease in intensity of the XRD reflections points to the formation of amorphous ${\text{Li}}_{2}S$ if jointly milled with LiI possibly serving as an additional grinding medium. The broad humps detected in the pattern support this interpretation. The broadening of the reflections is due to a decrease in crystallite size and the introduction of strain. Estimating the mean crystallite size with the help of the Scherrer equation^[^ ^39^ ^]^ yields a value of roughly 20 nm. Crystallite growth sets in upon annealing (or heat treatment, HT) and, consequently, the reflections become sharper again. Already after holding the sample for 3 h at 523 K or 623 K and higher, we estimate that the mean crystallite size reaches the μm range. The sample heat‐treated at 523 K is expected to show a mean crystallite ≥0.5 μm.

Independent of grain growth, even after the annealing step at 723 K (3 h), we do not re‐obtain exactly the same pattern as seen for the base mix. Therefore, either amorphous regions are still present or anion mixing is responsible for structural changes. Indeed, a magnification of the diffractogram by a factor of 5 clearly reveals the same broad humps as seen for the nanocrystalline sample, see the upper pattern in Figure 1a. However, even in the presence of amorphous fractions, we cannot exclude the formation of I‐containing ${\text{Li}}_{2}S$ regions. Indeed, careful inspection of the positions of the reflections shows that those of ${\text{Li}}_{2}S$ are slightly shifted, with respect to those of the base mix serving as a perfect reference, toward lower diffraction angles, see arrows. This slight shift suggests the formation of anion‐mixed LiI‐enriched lithium sulfide.^[^ ^40^ ^]^ Demixing is taking place upon annealing as the reflections shift back toward higher angles. In summary, the XRD patterns suggest the presence of at least three phases for nanocrystalline ${\text{Li}}_{2}S$‐LiI: amorphous ${\text{Li}}_{2}S$, nanocrystalline, defect‐rich or distorted LiI and LiI‐rich ${\text{Li}}_{2}S$ nanocrystals. The latter might also contain a larger amount of defects introduced through mechanical treatment. For comparison, in Figure 1c, the cubic crystal structures (anti‐fluorite type) of the two binaries are shown.

Alternatively, one might also think of incorporation of ${\text{Li}}_{2}S$ (Li on 8c and S on 4a) into LiI (Li on 4a and I on 4b); following this idea guides us to the study of Rajagopal et al.^[^ ^28^ ^]^ who assumed the formation of ${\text{Li}}_{3}\text{SI}$ crystallizing with antipervoskite‐like structure. In such a case, one might think of $S^{2-}$ occupying the 4*a* site in LiI; the additional Li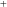 ions are expected on interstitial positions near S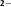 to ensure charge neutrality. Even in such a case, the intensity of the original LiI reflections might be increased. However, we do not see any significant shifts on the 2θ scale clearly supporting this hypothesis.

To shed more light on the structural changes, we recorded 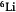 and 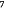
Li MAS NMR spectra that are shown in Figure 1b. The sample prepared by mixing the starting materials by hand shows two distinct and sharp resonances with characteristic chemical shifts for 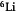 in ${\text{Li}}_{2}S$ (2.0 ppm) and LiI (−4.7 ppm), respectively. These shifts agree with those reported earlier.^[^ ^40^ ^]^ After ball milling and annealing, we essentially re‐obtain the original NMR spectrum; however, the NMR signal of LiI is somehow reduced in intensity. It might point to the fact that some I anions remain in the ${\text{Li}}_{2}S$ structure. We ensured full longitudinal relaxation of all spectral components and excluded $T_{1}$‐weighted resonances because this one‐pulse spectrum has been recorded after the sample was placed for 48 h in the external magnetic field, see also Experimental Section. The ball‐milled sample, in contrast, reveals broadened NMR signals centered at the same chemical shifts of the starting materials. Careful inspection reveals a distribution of resonances with very low intensity appearing between the 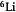 NMR signals of the starting materials, see arrow. This broad and featureless signal, see the magnification in Figure 1b, is suggested to reflect the Li spins subjected to anion‐mixed $S^{2-}$/I environments in ${\text{Li}}_{2}S$‐LiI nanocrystalline composites. Such environments would produce isotropic chemical shifts in between those of the unmilled starting materials as has been observed in detail for cation‐mixed (Ba,Ca)$F_{2}$ ^[^ ^41^ ^]^ (and other solid solutions such as (Pb,M)$F_{2}$ (M = Ba, Sr, Ca)) via 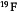 MAS NMR earlier.^[^ ^42^ ^]^ This broad NMR intensity is completely absent for both the base mix and the microcrystalline (annealed) sample. Most likely, anion‐mixed environments are found in the structurally disordered interfacial regions covering the nanocrystalline grains.

Alternatively, this broad resonance (Figure 1b) could also be understood as a signal arising from chemical exchange,^[^ ^43^, ^44^, ^45^ ^]^ that is, Li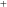 hopping between the magnetically inequivalent Li sites in environments of ${\text{Li}}_{2}S$ and LiI with negligible anion mixing. Such a coalescence effect involving two distinct crystallites^[^ ^40^, ^46^ ^]^ does, however, require the crystallographically different Li spins to be in close proximity to each other. The latter might be found for compacted, two‐phase nanocrystalline materials with a sufficiently high number of Li spins residing in the interfacial regions surrounding the nanocrystalline cores. The signal would then reflect a coalescence effect due to Li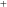 mass and spin transfer from one ${\text{Li}}_{2}S$ nanocrystallite to an adjacent LiI nanocrystal, as it has been suggested for the ${\text{Li}}_{2}\text{S(LiI)}-{\text{Li}}_{6}{\text{PS}}_{5}$Br interface by Wagemaker and coworkers.^[^ ^40^, ^46^ ^]^


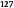
I MAS NMR (Figure 1c) reveals highly distorted environments the I spins are sensing. In general, for a structurally disordered or even amorphous phase, the NMR lines center around the same chemical shift values as seen for their crystalline and ordered counterparts. However, line broadening due to distortions and strain introduced leads to a distribution of chemical shifts producing a broadened resonance. Hence, while the crystalline compounds, that is, microcrystalline ${\text{Li}}_{2}S$‐LiI and LiI, reveal sharp resonances, that of ball milled ${\text{Li}}_{2}S$‐LiI produces a broad, asymmetric line even under the MAS conditions applied. It has to be noted that second‐order quadrupole effects cannot be removed with (ordinary) MAS NMR; hence, the asymmetry might be caused by quadrupolar effects to which the 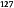
I quadrupole nucleus (I=5/2) is exposed. It points either to disordered single‐phase nanocrystalline LiI or to an anion‐mixed ${\text{Li}}_{2}S$‐LiI composite with cubic symmetry, as mentioned earlier.

### 2.2 Ion Dynamics as Probed by PCS and NSR

To understand ion dynamics in ${\text{Li}}_{2}S$‐LiI, we used spectroscopic techniques either taking advantage of electric or magnetic interactions of the mobile species with their surroundings. From the outset, these techniques are sensitive to translational motions of the Li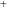 cations taking place on different timescales. Specifically, we used potentiostatic conductivity spectroscopy (PCS), 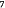
Li NMR line shape measurements, and 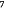
Li nuclear spin relaxation (NSR) to collect information on short‐range as well as long‐range ion dynamics.

#### 2.2.1 Potentiostatic Conductivity Spectroscopy

**Figure 2:**
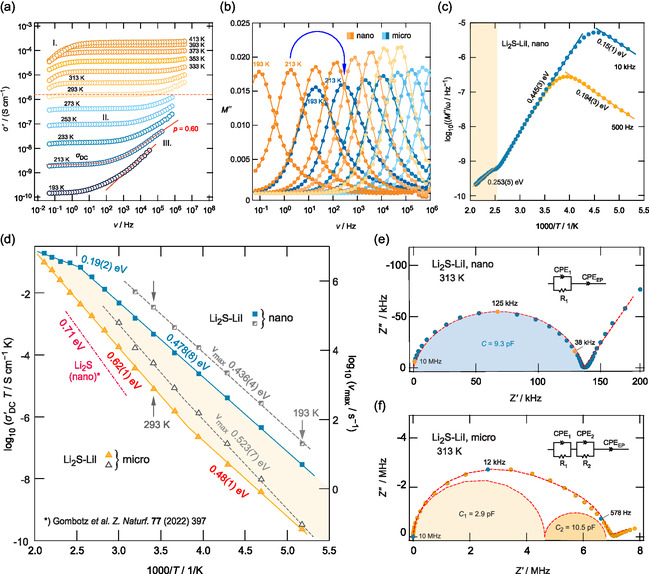
a) Conductivity isotherms σ′(ν) of the nanocrystalline ${\text{Li}}_{2}S$‐LiI sample recorded at the temperatures. Frequencies cover a range from 10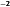 to 10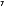 Hz. Solid lines show Jonscher fits containing the exponent *p*. b) Electric modulus peaks M″(ν) recorded at temperatures ranging from 193 to 473 K for both the nanocrystalline and the microcrystalline sample. The shift toward higher frequencies of the peak recorded at 213 K mirrors an increase in electrical relaxation frequency by two orders of magnitude. c) Change of the resistivity M″/ω of nanocrystalline ${\text{Li}}_{2}S$‐LiI recorded as a function of the inverse temperature but for constant frequency. Lines represent linear fits yielding the activation energies indicated. d) Arrhenius plot showing the temperature dependence of ${\sigma \prime }_{\text{DC}}$ and $\nu_{\text{max}}$ of the nano‐ and microcrystalline samples. Again, dashed and dotted lines represent linear fits. e) and f) Nyquist plots to analyze ‐Z″(Z′) of nanocyrstalline and microcrystalline ${\text{Li}}_{2}S$‐LiI. Electric equivalent circuits used to parameterize the whole responses are shown in the insets; capacitances in the pF range verify that bulk processes are probed.

In **Figure** **2**a, the conductivity isotherms of nanocrystalline ${\text{Li}}_{2}S$‐LiI are shown which are composed of the three classical regions as expected for a material with a single, overall conduction process with a distribution of electrical relaxation rates. At low *T*, the isotherms, constructed by plotting the real part of the complex conductivity σ′ against the frequency *ν*, consist of a frequency‐independent plateau (II.) and a dispersive region (III.) that follows Jonscher's power law, $\sigma \prime \propto \nu^{p}$. *p* values of 0.6 point to 3D ionic transport.^[^ ^47^, ^48^ ^]^ Region I., which is only seen at higher temperature, see Figure 2a, reflects piling up of the mobile Li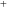 ions because of polarization effects in front of the ion‐blocking metal electrode used to record the isotherms.

From region II., we read off σ′(ν→0) values that describe long‐range ionic transport in ${\text{Li}}_{2}S$‐LiI; in this sense, σ′(ν→0) can be understood as “direct current” (DC) conductivity ${\sigma \prime }_{\text{DC}}$ to distinguish it from σ′ values of the dispersive regime III. collecting the “alternating current” (AC) response. At room temperature, we obtain ${\sigma \prime }_{\text{DC}}=1.6\times 10^{-6\;}{\text{S cm}}^{-1}$. The change of ${\sigma \prime }_{\text{DC}}$ is evaluated in Figure 2d in the frame of an Arrhenius diagram. According to ${\sigma \prime }_{\text{DC}}T\propto \exp(-E_{a}/(k_{B}T))$, where $k_{B}$ denotes Boltzmann's constant, we obtain an activation energy of ≈0.48 eV for the heating run. At higher temperatures, the Arrhenius line deviates from this linear behavior and passes into a line with a lower slope yielding an apparent activation energy of only 0.19 eV. Most likely, in situ annealing inside the impedance cell causes grain growth and healing of thermally nonresistant defects^[^ ^49^ ^]^ in both the interfacial regions and the bulk regions. These processes may finally lead to the deviation observed. Indeed, for the cooling run, much lower conductivities were obtained which agree very well with those seen for the microcrystalline (annealed) sample, which are also included in Figure 2a.

To further characterize ion dynamics in nanocrystalline ${\text{Li}}_{2}S$‐LiI, we evaluated variable‐temperature electric modulus peaks M″(ν) and resistivities M″/ω=f(1/T), see Figure 2b,c.^[^ ^50^, ^51^ ^]^ The peaks M″(ν) measured at constant *T* appear at distinct frequencies. By evaluating these characteristic electrical relaxation frequencies, $\nu_{\text{max}}$, again in the frame of an Arrhenius diagram, see the right axis of Figure 2b, we end up with an activation energy $E_{a\text{,}\;M}$ of 0.436 eV. The difference between $E_{a\text{,}M}$ and $E_{a}$ from ${\sigma \prime }_{\text{DC}}$ points to a slight temperature dependence of the charge carrier density $N_{c}$ to which ${\sigma \prime }_{DC}$ is sensitive, details of this concept are explained elsewhere.^[^ ^52^ ^]^ Most importantly, the uniform shapes of ${\sigma \prime }_{\nu }$ and M″(ν) point to a single, overall transport process. Considering the width of the M″(ν) peaks, this process has to be regarded as a distribution of electrical relaxation processes with $\nu_{\text{max}}$ being the average relaxation frequency.^[^ ^53^ ^]^

Plotting M″/ω against the inverse temperature 1/T, see Figure 2b, gives rise to asymmetric resistivity peaks whose position on the temperature scale shifts toward higher values the higher the frequency used to sample the data.^[^ ^50^, ^51^ ^]^ Here, we recorded M″/ω data at two frequencies, namely ω/2π=10 and 500 Hz. The high‐*T* sides coincide and yield an activation energy (0.445 eV) which is close to that extracted from electric modulus spectroscopy $E_{a\text{,}M}$. Again, at higher temperatures, we observe a deviation from the high‐*T* flank, which we ascribe to in situ annealing effects. Importantly, while the high‐*T* flanks of the isofrequent M″/ω(1/T) peaks probe long‐range ion transport, the low‐*T* flanks, just as σ′ in the AC regime III., are sensitive to short‐ranged ion dynamics, possibly including forward–backward hopping processes with much lower activation energies of 0.15 to 0.19 eV depending on *ω*. This asymmetry clearly points to a distribution of relaxation frequencies originating, most likely, from a highly heterogeneous potential landscape to which the hopping ions are subjected in structurally disordered, nanocrystalline ${\text{Li}}_{2}S$‐LiI. Taken together, this landscape produces regions II. and III. of the σ′(ν) conductivity isotherms. Depending on the exact timescale used to capture electrical relaxation phenomena, the activation energies range from 0.45 to 0.15 eV. A similar situation would be met in glassy materials with an irregularly formed potential landscape.^[^ ^54^ ^]^

While M″ and, thus, M″/ω are sensitive to bulk electrical relaxation, ${\sigma \prime }_{\text{DC}}$ might be influenced by grain boundary effects that could block long‐range ion transport. Here, evaluating the associated impedance data ‐Z″=f(Z′) in the Nyquist plane, we obtain a single, depressed semicircle that could be well approximated with a $R_{\text{1}}-{\text{CPE}}_{\text{1}}$ equivalent circuit, that is, a resistor R connected in parallel with a constant phase element (CPE), see Figure 2e. The spike in Figure 2e represents electrode polarization (EP) and is taken into account with a separate ${\text{CPE}}_{\text{EP}}$, see inset. The low capacitance associated with ${\text{CPE}}_{\text{1}}$ (9.3 pF) unequivocally reveals that ‐Z″=f(Z′), and, therefore, ${\sigma \prime }_{\text{DC}}$, reflects a bulk process.^[^ ^55^ ^]^

For comparison, the same conductivity analysis has been carried with the annealed sample. In Figure 2d, we included the change of ${\sigma \prime }_{\text{DC}}T(1/T)$ and $\nu_{\text{max}}(1/T)$ of microcrystalline ${\text{Li}}_{2}S$‐LiI. In contrast to nanocrystalline ${\text{Li}}_{2}$‐LiI, ${\sigma \prime }_{\text{DC}}T$ of microcrystalline ${\text{Li}}_{2}$‐LiI can be best understood in terms of two Arrhenius regimes yielding activation energies of 0.48 eV and 0.68 eV, as indicated. Similarly, two bulk relation processes are revealed in the Nyquist curves; the one belonging to 313 K is shown in Figure 2f. These two relaxation processes are less obvious if we analyze $\nu_{\text{max}}(1/T)$, see also Figure 2d. Independent of the exact nature of the two conduction processes seen in annealed ${\text{Li}}_{2}S$‐LiI, its overall ionic bulk conductivity is by almost two orders of magnitude lower than that probed for nanocrystalline ${\text{Li}}_{2}S$‐LiI. Ionic conductivity in nanocrystalline ${\text{Li}}_{2}S$‐LiI turned to be much higher than those of nanocrystalline ${\text{Li}}_{2}S$ thin films.^[^ ^11^, ^12^ ^]^ A lower conductivity has, however, been reported for nanocrystalline ${\text{Li}}_{2}S$ by some of us earlier.^[^ ^10^ ^]^ As mentioned earlier, structurally disordered interfacial regions, defect‐rich bulk regions, and nontrivial nanosize effects including space charge zones might contribute to this enhancement seen.^[^ ^29^, ^30^, ^31^, ^33^, ^36^, ^37^ ^]^ To shed light on the question if this clear increase of ionic transport of ${\text{Li}}_{2}S$‐LiI is also seen by 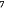
Li NMR spectroscopy, we carried out time‐domain NMR relaxation and line shape measurements.

#### 2.2.2 Nuclear Spin Relaxation

**Figure 3:**
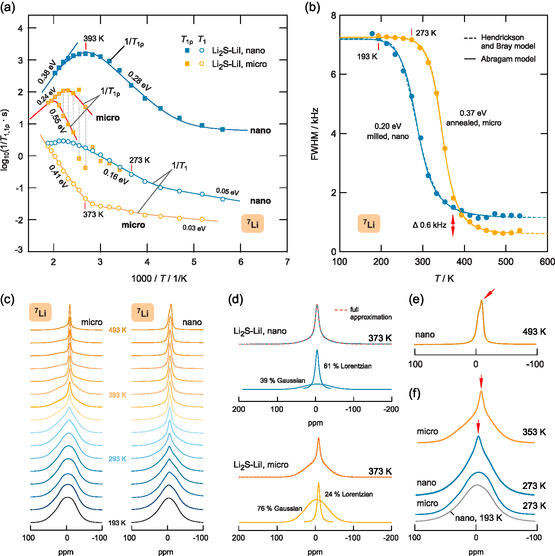
a) 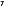
Li NMR relaxation rates recorded in both the laboratory ($1/T_{1}$, 116 MHz) and rotating frame of reference ($1/T_{1\rho }$, 20 kHz) of nanocrystalline and microcrystalline ${\text{Li}}_{2}S$‐LiI. The lines are to guide the eye or represent linear fits to extract the activation energies shown. b) Motional narrowing curves of the two samples; dashed and solid lines represent fits according to the models indicated in the key. c) 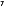
Li NMR line shapes of ball‐milled and annealed ${\text{Li}}_{2}S$‐LiI (100 ppm correspond to 11.64 kHz). d) Deconvolution of the NMR lines indicated (373 K, micro vs nano ${\text{Li}}_{2}S$‐LiI) with a combination of Gaussian and Lorentzian profiles. e) Comparison of selected 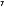
Li NMR lines of the two samples to magnify the evolution of the narrow line on top of the broad resonance. At 493 K, annealing inside the NMR sample chamber causes the appearance of an additional narrow line (the arrow is pointing at), which reflects the formation of microcrystalline ${\text{Li}}_{2}S$‐LiI. f) Selected NMR lines as indicated to compare the appearance of the narrow line on the ppm (or frequency) scale.

Starting with the nanocrystalline ${\text{Li}}_{2}S$‐LiI sample, we see that the $1/T_{1}$ 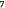
Li NMR spin‐lattice relaxation (SLR) rates if analyzed in the Arrhenius plot of **Figure** **3** show a weaker‐than‐activated temperature dependence below 250 K.

In this temperature range, SLR is mostly determined by lattice vibrations and interactions of the Li spins with paramagnetic impurities.^[^ ^56^, ^57^, ^58^ ^]^ At T>250 K, the rates pass into the low‐*T* flank of a relatively broad but diffusion‐induced rate peak $1/T_{1}(1/T)$.^[^ ^56^, ^57^, ^58^, ^59^ ^]^ Its activation energy in the limit $\tau_{c}\omega_{0}=1$, with $\tau_{c}$ being the motional correlation time and $\omega_{0}/2\pi$ denoting the Larmor frequency, amounts to only 0.16 eV and agrees with those seen in resistivity spectroscopy, see Figure 2c. Hence, the rates are governed by short‐range motional processes. Importantly, the rates measured above 400 K might already be influenced by annealing effects, as also seen in conductivity spectroscopy, as mentioned earlier.

Turning to the rates $1/T_{1\rho }$ measured in the rotating frame of reference, that is, at much lower values of $\omega =\omega_{1}<\omega_{0}$, hopping processes on a longer timescale are probed.^[^ ^56^ ^]^ In the case of a distribution of correlation times, a larger activation energy (0.28 eV) governs the low‐*T* side of the peak. The spin‐lock peak appears, as expected, at a lower temperature to obey the condition $\tau_{c}\omega_{0(1)}\approx 1(0.5)$.^[^ ^56^, ^57^, ^60^ ^]^ On the high temperature side, we expect an activation energy that captures true long‐range ion dynamics.^[^ ^56^ ^]^ Indeed the few rates accessible in this regime lead to a value of ≈0.38 eV (see Figure 3a), which is in fair agreement with those probed by ${\sigma \prime }_{\text{DC}}T$, $\nu_{\text{max}}$, and M″/ω, as mentioned earlier. Again, we have to consider that these rates might already be influenced by annealing effects taking place inside the NMR sample chamber when the sample is kept at such high *T* during the measurements. In general, any asymmetry of a given $1/T_{1\rho }(1/T)$ peak is reflected by the parameter *β*, which can be used to modify the original Lorentzian‐type spectral density function $J(\omega_{0})$ used to describe the temperature and frequency dependence of $1/T_{1\rho }\propto \tau_{c}/(1+{\left(\tau_{c}\omega_{1}\right)}^{\beta }$).^[^ ^56^, ^60^, ^61^, ^62^ ^]^ Only for uncorrelated motion, β=2 is expected.^[^ ^56^ ^]^ Here, a value of β=0.38/0.28+1≈1.7 is obtained,^[^ ^57^, ^62^ ^]^ which points to correlated motion originating from the interplay of Coulomb interactions and structural disorder.^[^ ^56^, ^63^ ^]^

For comparison, in Figure 3a, we also included the corresponding $1/T_{1}$ 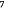
Li NMR SLR rates of microcrystalline ${\text{Li}}_{2}S$‐LiI. The shift of the respective flanks toward higher temperatures clearly shows a lower overall diffusivity. Interestingly, even for the low‐*T* flank of the $1/T_{1}(1/T)$ rate peak, a rather large activation energy of 0.41 eV is obtained. In agreement with the two conduction processes seen in impedance spectroscopy, the spin‐lock NMR transients show bi‐exponential behavior leading to two distinct rates $1/T_{1\rho }$ if T>360 K. From the two spin‐lock peaks shown in Figure 3a, we extract activation energies as high as 0.55 eV showing that NMR is able to sample even jump processes over large barriers in the annealed ${\text{Li}}_{2}S$‐LiI sample.

The obvious change in ion dynamics between the two samples becomes also evident if we look at the change of the width (full width at half maximum) of the 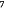
Li NMR spectra, see Figure 3b,c. Onset of motional line narrowing of nanocrystalline ${\text{Li}}_{2}S$‐LiI is shifted by 80 K toward lower temperature. The dashed and solid lines represent analyses of the shape of the narrowing curve with the models introduced by Abragam^[^ ^64^ ^]^ as well as by Hendrickson and Bray.^[^ ^65^ ^]^ In both cases, only mean values of activation energies are obtained. The conditions to which these models refer do only allow a rough estimation of activation energies in the case of complex ion conductors. The values of 0.2 and 0.37 eV obtained here might be compared with those of the low‐*T* flanks of the respective $1/T_{1}(1/T)$ rate peaks (≈0.16 and 0.41 eV). The larger line width of nanocrystalline ${\text{Li}}_{2}S$‐LiI in the regime of extreme narrowing (600 Hz, see Figure 3b) is ascribed to a broader distribution of chemical shifts in the ball‐milled sample.

The NMR line shapes shown in Figure 3c reveal details on the nature of the averaging process of homonuclear dipole–dipole interactions through the irregular Li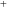 hopping processes. Already slightly below room temperature, the line of nanocrystalline ${\text{Li}}_{2}S$‐LiI is partly narrowed showing a tip on top of the broader Gaussian profile. Full narrowing is achieved at 400 K. In Figure 3d, we see that a combination of a Gaussian and a Lorentzian is needed to fully reproduce the overall line shape at 373 K. If assuming that quadrupole interactions play only a minor role in our cubic system here, the area fraction of the narrowed tells us that ≈60% of the ions participate in fast exchange processes at this temperature. In general, such two‐component lines are expected in the regime of motional narrowing if we deal with heterogeneous dynamics, i.e., a (broad) distribution of jump rates, frequently met in disordered systems.^[^ ^66^, ^67^ ^]^ For the microcrystalline sample, a similar picture is obtained; however, the emergence of the narrow component is shifted toward higher temperatures; at 373 K, the area under this line amounts to only 27%, see Figure 3d. We also note some off‐centering of the narrow line; in the case of nanocrystalline ${\text{Li}}_{2}S$‐LiI it is shifted toward positive ppm values, and in the case of the microcrystalline material toward negative ppm values, see Figure 3f. Lastly, these different shifts also explain the splitting of the 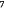
Li NMR line of nanocrystalline ${\text{Li}}_{2}S$‐LiI obtained at 493 K. At this temperature, an even narrower line emerges whose position agrees with that of the microcrystalline sample at the same temperature (Figure 3e). This observation reflects irreversible changes of the nanocrystalline sample if exposed to high temperatures, which have also been detected by conductivity spectroscopy, as mentioned earlier. Structural relaxation, grain growth, and defect healing are expected to take place at higher *T*, which finally convert the disordered nanocrystalline ${\text{Li}}_{2}S$‐LiI material into its structurally ordered (microcrystalline) counterpart.

To analyze the origin of faster ion exchange in the nanocrystalline sample that takes benefit from structural disorder, we used (high‐resolution (MAS)) variable‐temperature 1D and 2D 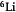 NMR to directly observe hopping of the ions between magnetically ineqeuivalent crystal sites.

#### 2.2.3 1D and 2D EXSY MAS NMR

**Figure 4:**
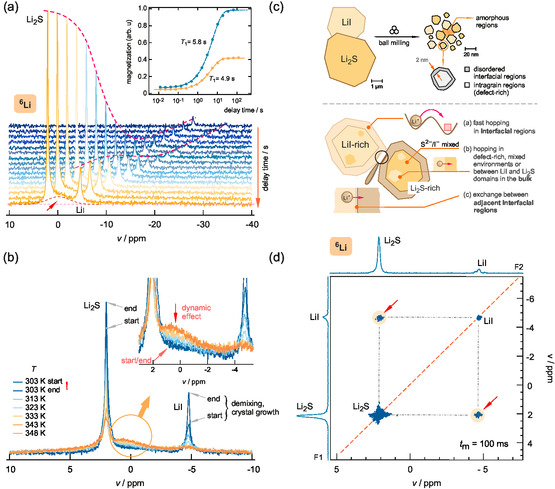
a) 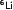 MAS NMR spectra of nanocrystalline ${\text{Li}}_{2}S$‐LiI (73.6 MHz) recorded in the frame of a saturation recovery SLR experiment under MAS conditions (25 kHz spinning frequency). The inset shows the resulting magnetization transients following slightly stretched exponentials and yielding site‐specific relaxation times $T_{1}$. b) Evolution of the coalesced 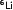 MAS NMR signal of nanocrystalline ${\text{Li}}_{2}S$‐LiI (see arrow in the inset) with increasing temperature. Lines have been normalized with respect to the area under the total NMR signal. We also notice a slight change of the main resonances due to in situ annealing this sample. c) Illustration of the structural picture as refined by high‐resolution 1D 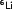 NMR in combination with X‐ray diffraction. Hopping processes can take in the interfacial (disordered, amorphous) regions, in the mixed bulk regions including hopping between different domains, and between closely adjacent interfacial regions of different crystallites. d) 2D 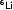 MAS EXSY NMR experiment (100 ms mixing time) to visualize hopping of 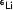 between the distinct magnetically sites in nanocrystalline ${\text{Li}}_{2}S$‐LiI.

**Figure** **4**a shows the 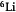 MAS NMR lines of nanocrystalline ${\text{Li}}_{2}S$‐LiI, recorded at a spinning speed of 25 kHz, as a function of recycle delay time $t_{d}$ that is varied in the frame of a saturation recovery experiment to measure the rate $1/T_{1}$. At short delay times, we already detect the resonance belonging to 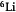 in an ${\text{Li}}_{2}S$ environment, see the line at 2 ppm.

With increasing $t_{d}$, the line characterizing 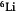 in the LiI environment emerges. At sufficiently long waiting times, the two signals get saturated and longitudinal recovery is finished, see the inset in Figure 4a showing the respective transients $M_{z}(t_{d})$. These were constructed by plotting the area under the individual NMR signal as a function of delay time. The resulting site‐specific relaxation times, obtained after analyzing the recovery curve with a stretched exponential, amount to 5.8 and 4.9 s. Almost the same $T_{1}$ time is obtained for the broad signal in between the resonances for ${\text{Li}}_{2}S$ and LiI of nanocrystalline ${\text{Li}}_{2}S$‐LiI. Obviously, no large difference in relaxation behavior is seen for the individual spectral NMR components of ball‐milled ${\text{Li}}_{2}S$‐LiI.

This result is in stark contrast to single‐phase nanocrystalline LiI and nanocrystalline ${\text{Li}}_{2}S$ that have been ball‐milled under the same conditions as the ${\text{Li}}_{2}S$‐LiI binary mixture. The 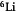 NMR $T_{1}$ time of nanocrystalline LiI turned out to be 35 s and that of nanocrystalline ${\text{Li}}_{2}S$ is estimated to be 2–3 orders of magnitude longer. The same holds for microcrystalline ${\text{Li}}_{2}S$‐LiI for which we estimate immensely long site‐specific 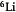 NMR relaxation times. Hence, the 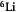 is a highly sensitive probe to verify that the two resonances of ball‐milled ${\text{Li}}_{2}S$‐LiI are exposed to almost the same relaxation behavior, which is i) completely different to that in single‐phase nanocrystalline LiI or ${\text{Li}}_{2}S$ and ii) no longer comparable to that in the structurally ordered microcrystalline reference. The increase in 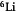 NMR $T_{1}$ time, observed when going from nanocrystalline LiI to ball‐milled ${\text{Li}}_{2}S$‐LiI, clearly reveals that a mixing effect of the two binaries is important to explain the change seen.

With this result, we can refine our picture of the ${\text{Li}}_{2}S$‐LiI nanocrystals prepared, see the illustration in Figure 4b. We expect anion mixing not only limited to the outer, disordered interfacial regions covering the nanocrystals but also, to a lesser extent, inside the crystalline grains. Fast exchange processes would give rise to shorter (site‐specific) relaxation times as compared to the unmixed samples. These would lead, with the assistance of fast spin‐diffusion rates on the Hz timescale, to a uniform spin temperature and, thus, to very similar $T_{1}$ times. In general, such flip‐flop processes will involve spins in fast longitudinal relaxation that are even farther away from a $S^{2‐}$/I mixed environment.

To definitely visualize Li exchange between the two sharp resonances in Figure 4a, we carried out 2D 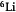
‐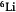 MAS exchange spectroscopy (EXSY) experiments,^[^ ^40^, ^43^, ^46^ ^]^ see Figure 4d for a 2D MAS EXSY experiment recorded at a mixing time $t_{m}$ of 100 ms. This mixing time is much shorter than $T_{1}$; hence, we can safely rule out any artifacts from longitudinal spin‐lattice relaxation effects. Off‐diagonal intensities appear if during $t_{m}$ frequency, fluctuations $\omega (t_{0})\neq \omega (t_{m})$ take place that directly originate from translational displacements of the 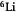 spins. Cross peaks due to spin‐diffusion effects are also ruled out because in a sample with natural abundance of 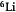 (7.5%), we deal with a diluted spin system that reduces homonuclear coupling through the spatially separated spins.^[^ ^68^ ^]^ For $t_{m}>80\;\text{ms}$, the appearance of cross peaks starts and their intensity increases with $t_{m}$. In contrast, no off‐diagonal intensities are observed for the microcrystalline sample even if we record spectra at mixing times $t_{m}$ as long as 500 ms. As an example, the corresponding 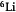 MAS EXSY NMR data of the microcrystalline sample, exhibiting no off‐diagonal intensities ($t_{m}=100\;\text{ms}$), is shown in Figure S1, Supporting Information. Here, 2D 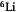 MAS EXSY of the nanocrystalline mixture unequivocally shows 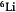
‐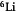 exchange between, in our opinion, neighboring Li ions in the two environments ${\text{Li}}_{2}S$ and LiI.

Lastly, we studied the temperature behavior of the broad NMR signal between the distinct 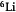 resonances of ${\text{Li}}_{2}S$ and LiI, see Figure 4c. Heating the sample inside the MAS rotor from 303 K to 348 K clearly shows that this signal increases in intensity, while the outer signals of ${\text{Li}}_{2}S$ and LiI loose in height. Since the lines coalesce and do not narrow individually, this behavior is attributed to a classical exchange process^[^ ^44^, ^45^ ^]^ setting in if the exchange rate reaches the spectral distance of the two participating signals (≈800 Hz). This result fully supports our findings from 2D EXSY NMR sensing this process on a longer timescale defined by $1/t_{m}=10\;\text{Hz}$. Line coalescence is fully reversible, as the coalesced line looses in intensity when going back to 303 K, see the spectra labeled with start and end. Slight changes of the main resonances might indicate the beginning of defect healing and demixing occurring in situ inside the MAS rotor when exposed to 348 K for a sufficiently long time. In summary, from both variable‐temperature 1D 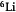 MAS NMR and 2D 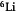 MAS EXSY NMR, we find strong evidences that Li ions hop between adjacent Li sites in anion‐mixed, nanocrystalline ${\text{Li}}_{2}S$‐LiI, which might be composed of ${\text{Li}}_{2}S$‐rich and LiI‐rich domains. We think that S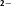/I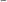 mixed sites or domains can be found to a large extent in the disordered interfacial regions of ball‐milled ${\text{Li}}_{2}$‐LiI. As mentioned earlier, these hops might even reflect exchange between Li sites in different interfacial regions.^[^ ^40^, ^46^ ^]^ In addition, mixed sites in the crystalline grains will contribute to the overall enhanced diffusivity too (see Figure 4b).

## 3 Conclusion

${\text{Li}}_{2}S$ is the final discharge product in Li–sulfur batteries. In its nanocrystalline form, it shows enhanced ion dynamics. The influence of LiI on ion dynamics, here realized by mechanical treatment of a ${\text{Li}}_{2}S$‐LiI mixture, was studied by X‐ray diffraction, broadband conductivity spectroscopy as well as time‐domain and high‐resolution 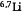 NMR spectroscopy. Compared to single‐phase ${\text{Li}}_{2}S$, electrical conductivity measurements clearly reveal a further increase of overall bulk ion dynamics by two orders of magnitude. This increase might be of interest in Li–sulfur battery technology if it would be possible to create nanostructured I‐bearing ${\text{Li}}_{2}S$ as the desired discharge product. Here, 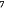
Li SLR NMR measurements support the findings of enhanced Li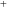 diffusivity and point to heterogeneous dynamics in mixed ${\text{Li}}_{2}S$‐LiI, that is, a distribution of jump rates, at least on the NMR timescale. Altogether, activation energies for long‐range and short‐range ion dynamics range from 0.16 to 0.48 eV in nanocrystalline ${\text{Li}}_{2}S$‐LiI.

Combining the results from X‐ray diffraction with those from high‐resolution 1D and 2D 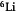 MAS NMR, we found evidence that, apart from amorphous regions produced, anion‐mixed environments are formed that allow facile Li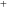 exchange between magnetically inequivalent Li ions in the ${\text{Li}}_{2}S$ and LiI environments. The latter has been verified by 2D 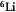 EXSY NMR and variable‐temperature 1D 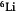 MAS NMR spectra showing off‐diagonal intensities and coalescence effects upon heating the sample. Hence, the interaction between the two binaries significantly increases Li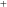 ion dynamics ($1.6\times 10^{-6}\;S\;{\text{cm}}^{-1}$), provided ${\text{Li}}_{2}S$‐LiI is kept in its original nanocrystalline state directly obtained after high‐energy ball milling.

## 4 Experimental Section

4.1

4.1.1

##### Sample Preparation

The various ${\text{Li}}_{2}S$‐LiI samples were prepared by joint high‐energy ball mill (Fritsch Pulverisette 7 premium line) of the starting materials; subsequent annealing steps yielded the samples investigated in this study. ${\text{Li}}_{2}S$ (Sigma Aldrich 99,98%) and LiI (Sigma‐Aldrich, 99.999%) were mixed in a molar ratio of 1:1, if not stated otherwise, and were transferred in a ${\text{ZrO}}_{2}$ milling beaker (45  mL). The vial was filled with 180 ${\text{ZrO}}_{2}$ balls. The mixed powder was milled for 5 min followed by a 10 min break; the milling speed was 600 rpm. We used a total of 120 cycles to prepare nanocrystalline ${\text{Li}}_{2}S$‐LiI. In addition, parts of this powder were subjected to different annealing temperatures ranging from 423 up to 723 K; the samples were kept at these temperatures for 3 h each. For these annealing steps, the ball‐milled powder was uniaxially pressed into pellets (5 m in diameter) using a load of 0.5 tons. The sample obtained after an annealing step at 723 K is called the microcrystalline sample, whereas the sample prepared without any annealing step is referred to as nanocrystalline sample. All preparation steps were carried out either in Ar atmosphere with <1 ppm $H_{2}O$ and <1 ppm $O_{2}$, respectably, or under vacuum. The starting materials were used as received without applying any further purification steps.

##### X‐Ray Diffraction (XRD)

XRD measurements were carried out using a Rigaku Miniflex 600 diffractometer operating with Bragg Brentano geometry (Cu $K_{\alpha }$ radiation) in combination with a D/teX Ultra silicon strip detector. The patterns were recorded covering a 2θ range from 10° to 60° using a step size of 0.02°. To avoid contamination of the powders with air or moisture, an air‐tight sample holder was used. The results were analyzed using Malvern Panalyticals X’PertHighScorePlus software.

##### Conductivity Spectroscopy

Broadband conductivity measurements were carried out with a Novocontrol Concept 80 broadband dielectric spectrometer covering frequencies form *ν* = 10 mHz up to 10 MHz. The temperatures were varied from 193 to 473 K ± 1 K with a QUARTO cryosystem (Novocontrol) using freshly evaporated dinitrogen $N_{2}$ and a heater to automatically adjust the temperature in the sample chamber. For these measurements, an in‐house built air‐tight sample holder was used to prevent contaminations with air and moisture of the pellets. Prior to the measurements, we prepared dense pellets of the samples using a hand press placed inside an Ar‐filled glove box. Au blocking electrodes were applied by taking advantage of a Leica EM SCD 050 sputter coater, which was used to apply a 50 nm Au layer on each side of the pellet. The sputter coater was placed inside the Ar glove box to eliminate any contamination with moisture.

##### Nuclear Magnetic Resonance (NMR):NMR Relaxation

For the static NMR experiments, the powder samples were sealed in Duran glass tubes under vacuum to prevent them from any further contact with air or even moisture. Solid‐state NMR measurements under static conditions were performed to investigate the change of line shapes and the evolution of SLR rates containing information on jump rates and activation energies. The variable‐temperature SLR NMR measurements were carried out in the laboratory frame of reference (1/$T_{1}$) and in the rotating frame of reference (1/$T_{1\rho }$, see below) using a Bruker Avance III spectrometer connected to a shimmed cryomagnet with a nominal magnetic field of 7 T. We covered a temperature range from 178 to 533 K. For all measurements, a ceramic high‐temperature probe (Bruker) was employed. For the 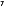
Li SLR NMR measurement, the well‐known saturation pulse sequence was used to acquire the (diffusion‐induced) recovery of the longitudinal magnetization $M_{z}(t)$.^[^ ^69^, ^70^ ^]^ The magnetization transients $M_{z}$($t_{d}$) describing longitudinal spin relaxation were constructed by plotting the area under the free induction decays (FIDs) against the variable waiting time $t_{d}$. Sixteen FIDs were accumulated for each waiting time. The transients $M_{z}$($t_{d}$), consisting of 16 data points, were analyzed with stretched exponentials, $M_{z}(t_{d})\propto 1-\exp(-{\left(t_{d}/T_{1}\right)}^{\gamma }$), where *γ* (0<γ≤1) is the stretching coefficient to take into account deviations from ideal exponential time behavior (γ=1).

Furthermore, we conducted NMR spin‐lattice experiments in the so‐called rotating frame of reference using the spin‐lock technique.^[^ ^69^ ^]^ Here, we used a locking pulse corresponding to a frequency $\omega_{1}/2\pi$ of 20 kHz. The resulting transversal magnetization transients $M_{\rho }(t_{\text{lock}})$ were analyzed similarly as for the measurements performed in the laboratory frame by parameterizing the area under the FIDs with stretched exponentials. Whenever a stretched exponential function proved to be insufficient to reproduce the curves, we used a double exponential function to analyze the data: $M_{z(\rho )}(t)\propto \exp(-(t/T_{1(\rho ),1}))+\exp(-(t/T_{1(\rho ),2}))$. Indeed, for temperatures ranging from 373 to 473 K, the transients for the *micro*crystalline sample had to be fitted with this ansatz to ensure a good quality of the fit. The resulting 1/$T_{1(\rho )}$ rates were extracted and plotted in an Arrhenius diagram to derive activation energies and jump rates.

##### Nuclear Magnetic Resonance (NMR): High‐Resolution NMR

MAS NMR spectra of the nuclei 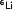 (73.6 MHz; spin‐quantum number I=1/2) and 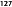
I (100.1 MHz; I=5/2) MAS were recorded with a Bruker Avance spectrometer in connection to a shimmed magnet with a nominal magnetic field of 11.7 T. The powder samples were filled in 2.5 mm‐zirconia MAS rotors constructed to be exposed to spinning speeds of 25(1) kHz. For the microcrystalline sample, the 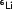 MAS NMR lines at ambient bearing gas temperature were recorded with a single scan after placing the rotor in the magnetic field for a period of 48 h (172 800 s). This approach ensured the full establishment of the distribution of the spins over the two Zeeman levels according to the Boltzmann equilibrium at this *T*. The same saturation was ensured for the nanocrystalline sample. The so‐obtained FIDs were Fourier‐transformed without further manipulation of the time‐domain signal. Solid ${\text{CH}}_{3}\text{COOLi}$ (0.1 ppm vs 0 ppm of 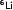 in LiCl aqueous solution) served as secondary reference to determine the NMR chemical shifts $\delta_{\text{iso}}$. 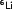 MAS NMR line of the nanocrystalline sample were also recorded for a series of temperatures, that is, from ambient to 348 K. In addition, we also measured $1/T_{1}$ NMR rates under MAS conditions to collect site‐specific information on longitudinal relaxation.

##### Nuclear Magnetic Resonance (NMR): 2D EXSY NMR

Finally, 2D EXSY NMR data were recorded by using nuclear Overhauser effect spectroscopy (NOESY) experiments. 2D EXSY NMR spectra (16 scans, dwell time of 50 μs) were recorded at ambient bearing gas temperature. Rotation synchronized acquisition was performed in both F2 (4096 points) and F1 (12 points) directions; states‐TPPI (time‐proportional phase incrementation) for quadrature detection in F1 and phase correction in F1 direction were employed. The mixing time of the EXSY experiments varied from 50 ms up to 600 ms.

## Conflict of Interest

The authors declare no conflict of interest.

## Author Contributions

**Anna Jodlbauer**: Data curation (lead); Formal analysis (equal); Investigation (equal); Methodology (equal); Writing—original draft (equal); Writing—review and editing (equal). **Katharina Hogrefe**: Conceptualization (supporting); Formal analysis (supporting); Investigation (supporting); Supervision (equal); Writing—original draft (supporting). **Bernhard Gadermaier**: Conceptualization (supporting); Formal analysis (equal); Methodology (equal); Supervision (supporting); Writing—original draft (supporting); Writing—review and editing (supporting). **H.** **Martin R. Wilkening**: Conceptualization (lead); Formal analysis (equal); Funding acquisition (equal); Investigation (equal); Methodology (equal); Project administration:(equal); Resources:(equal); Supervision:(equal); Writing—original draft:(supporting); Writing—review and editing: (lead).

## Supporting information

Supplementary Material

## Data Availability

The data that support the findings of this study are available from the corresponding author upon reasonable request.
