# Supplementary material for: Ion Dynamics in Nanocrystalline Li2S‐LiI – on the Influence of Local Disorder on Short‐Range Hopping and Long‐Range Ion Transport
Source: Small Sci. 2024 Jul 30;4(10):2400199. doi: 10.1002/smsc.202400199 (PMC11935101; doi:10.1002/smsc.202400199)
Supplement: Supplementary file 1 — Supplementary Material [file SMSC-4-2400199-s001.pdf]

Supporting Information

Ion dynamics in nanocrystalline Li_2S-LiI – on the influence of local disorder on short-range hopping and long-range ion transport

Anna Jodlbauer, Katharina Hogrefe, Bernhard Gadermaier, and

H. Martin R. Wilkening*

Institute of Chemistry and Technology of Materials, Graz University of Technology, Stremayrgasse 9, A-8010 Graz, Austria

E-mail: wilkening@tugraz.at

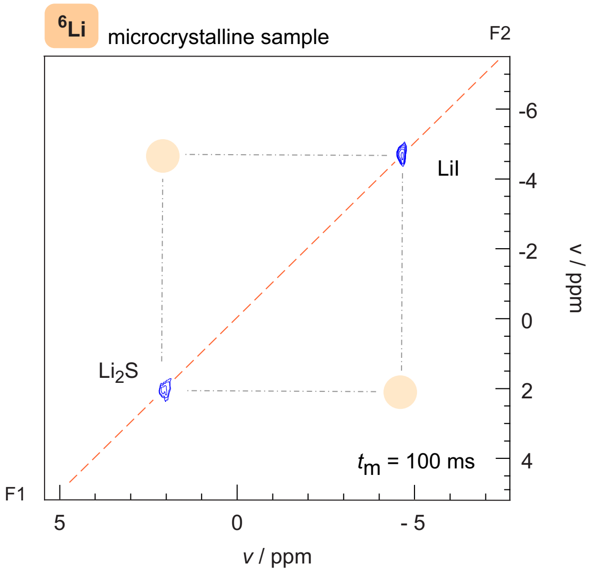

Figure S1: 2D ^{6}Li MAS EXSY NMR experiment to check whether a direct exchange between the magnetically distinct Li_2S and LiI sites is occurring in microcrystalline Li_2S-LiI.

In Figure S1 the 2D ^{6}Li MAS EXSY NMR experiment of micryocrystalline Li_2S-LiI is shown that has been recorded with a mixing time of 100 ms. The yellow regions in the graph indicate the positions for possible off-diagonal cross peaks. At mixing times as long as 100 ms, no such peaks are, however, detected. Hence, exchange processes in the microcrystalline sample proceed on a much slower time scale as compared to those in the nanocrystalline counterpart, see Figure 4d.
